# Albuminocytologic Dissociation and Intravenous Immunoglobulin Therapy in Parsonage-Turner Syndrome With Bilateral Involvement: A Case Report

**DOI:** 10.7759/cureus.80342

**Published:** 2025-03-10

**Authors:** Kevin Szafran, Justin Wang, Leslie Wong, Forrest Butensky, Kanwardeep Singh

**Affiliations:** 1 Medical School, American University of the Caribbean School of Medicine, Cupecoy, SXM; 2 Physical Medicine and Rehabilitation, Nassau University Medical Center, East Meadow, USA; 3 Interventional Pain Medicine, Rutgers University New Jersey Medical School, Newark, USA

**Keywords:** ascending flaccid paralysis, brachial plexopathy, electromyography (emg), emg-ncv, multidisciplinary care, neurology case report, off-label drug use, parsonage-turner syndrome, physical medicine and rehabilitation, target protein biomarkers csf

## Abstract

Parsonage-Turner syndrome (PTS) is a rare neurological disorder characterized by acute neuropathic pain followed by motor and sensory deficits, typically affecting the brachial plexus. While often self-limiting, atypical presentations can complicate diagnosis and management. We present a case of a 53-year-old male patient with a history of cervical foraminal stenosis and progressive left upper extremity (LUE) flaccid paralysis for over 14 months, with no clear cause for worsening symptoms. Diagnostic evaluation, including magnetic resonance imaging (MRI) and computed tomography (CT) of the brain, cervical spine, and brachial plexus, revealed grossly normal findings. Initial electromyography (EMG) studies demonstrated worsening motor response in the LUE without the typical dermatomal distribution of cervical radiculopathy, leading to the diagnosis of PTS. Additionally, shoulder subluxation and triceps tendon insertional enthesopathy were noted due to muscular instability. The patient was followed by outpatient neurology and physical medicine and rehabilitation departments when, 14 months later, he developed similar weakness in the contralateral right upper extremity (RUE). In the outpatient clinic, repeat EMG demonstrated severe axonal denervation with no motor or sensory response in the LUE, along with new-onset RUE weakness in the digits, prompting hospital admission. During a week-long hospitalization, all blood tests were normal, and infectious causes were ruled out. Notably, cerebrospinal fluid (CSF) analysis revealed albuminocytologic dissociation (ACD), a unique finding in the patient with a history of PTS. Given the conflicting presence of ACD and the potential for an underlying autoimmune inflammatory neuropathy, the patient was treated off-label with intravenous immunoglobulin (IVIG). IVIG was selected over corticosteroids due to the chronic and worsening nature of the condition, as there is limited clinical evidence supporting steroid efficacy in long-term cases. Physical therapy was initiated during hospitalization, leading to modest improvement in the RUE motor strength. This case highlights the diagnostic challenges of PTS, particularly in patients with bilateral involvement, atypical progression, and severe symptoms. A multidisciplinary approach, including the exclusion of other neuromuscular and structural pathologies, is essential. Early recognition and intervention may help mitigate long-term morbidity. Further research into early diagnostic markers and targeted treatments is warranted to improve patient outcomes and restore neuromuscular function.

## Introduction

Parsonage-Turner syndrome (PTS), also known as idiopathic brachial plexopathy, acute brachial neuropathy, brachial plexus neuritis, shoulder girdle syndrome, neuralgic amyotrophy, or paralytic brachial plexus and acute brachial radiculitis, is a rare neurological disorder characterized by the sudden onset of severe, disproportionate pain in the shoulder and upper arm, followed by muscle weakness, atrophy, and, in some cases, sensory deficits. With an estimated prevalence of three in 100,000 individuals, PTS is uncommon but clinically significant [1]. Specific nerves within the brachial plexus may be variably affected, and the clinical presentation often mimics other conditions.

The exact etiology of PTS remains incompletely understood, though it is frequently triggered by identifiable factors such as viral or bacterial infections, recent vaccinations, physical trauma, or autoimmune phenomena [2]. Genetic research has also identified mutations in the SEPTIN-9 gene, which regulates cell cycle control and cytokinesis in nerve cells, suggesting that hereditary predisposition may contribute to certain cases, particularly those with recurrent and more frequent episodes [3].

Historically referred to as "acute idiopathic brachial neuritis," PTS was first described over a century ago in patients presenting with sudden-onset upper extremity pain and subsequent flaccid paralysis following influenza infection [4]. Other studies have linked a correlation of development of PTS to shingles vaccines, surgical complications, viral infections (varicella virus, herpes simplex virus, HIV, coxsackie B virus, hepatitis B virus, hepatitis C virus, Epstein-Barr virus, cytomegalovirus, SARS-CoV2), and autoimmune and systemic disease; however, many cases are idiopathic in nature. The hallmark feature of PTS is severe pain and weakness that is often disproportionate to physical findings. Unlike many musculoskeletal conditions, this pain is not exacerbated by positional changes, neck movements and compression, an insidious onset, and does not follow a dermatomal pattern, but frequently worsens at night, leading to sleep disturbances. Sensory abnormalities such as allodynia, paresthesia, or hyperesthesia are common, alongside muscular weakness, flaccidity, and progressive atrophy [5].

PTS predominantly affects the upper trunk of the brachial plexus, with frequent involvement of the suprascapular, axillary, and musculocutaneous nerves. This can result in functional deficits such as impaired shoulder abduction, external rotation, and elbow flexion. Lower plexus involvement may affect the ulnar, radial, and median nerves, leading to dysfunction in distal upper extremity and hand muscles. Rarely, involvement of the phrenic or long thoracic nerves can result in atypical presentations such as diaphragmatic paralysis or scapular winging, further complicating the diagnosis. Most cases tend to be unilateral in nature, and some have shown to progress bilaterally [6].

Despite advances in diagnostic techniques, PTS remains a clinical challenge due to overlapping features with cervical radiculopathy, rotator cuff pathology, stroke-like symptoms, nervous system infections, and other neuromuscular disorders. It is considered a diagnosis of exclusion, and the clinical course is highly variable. While the condition is often self-limiting, with up to 90% of patients regaining significant muscle strength within three years, many experience residual deficits, including persistent paresis, chronic pain, amyotrophy, or sensory abnormalities [7]. These prolonged symptoms can lead to significant functional impairments and diminished quality of life, underscoring the need for early recognition and a multidisciplinary approach to management.

After a thorough clinical evaluation and laboratory testing, electromyography (EMG) can help identify nerve distributions affected by PTS, support clinical findings, and rule out other pathologies. EMG in PTS typically reveals a patchy pattern of denervation across multiple nerves and spinal root levels, reflecting multifocal involvement of the brachial plexus or its branches. Magnetic resonance imaging (MRI) can also aid in diagnosis by showing muscle hyperintensities and nerve hourglass constrictions, which can also be detected via ultrasound with 92% sensitivity. EMG has a sensitivity of approximately 96.3% for detecting axonal denervation, but 3.7% of patients may show no abnormalities, especially if tested within three weeks of symptom onset. These diagnostic tests show clear efficacy in some populations; however, early-stage PTS may not always present with clear nerve conduction and imaging abnormalities, limiting its diagnostic reliability. These findings highlight the usefulness of EMG and MRI in diagnosing PTS, while also emphasizing their limitations, particularly in early or atypical presentations. Initial pain crises can be managed with analgesics, oral corticosteroids, and immobilization, while chronic cases benefit from physical therapy combined with co-analgesic strategies for symptomatic relief. In select cases, particularly those with suspected autoimmune or inflammatory origins, off-label intravenous immunoglobulin (IVIG) has shown potential benefits. However, IVIG administration carries significant risks, including anaphylaxis, thrombosis, acute kidney injury, and cardiovascular effects, necessitating careful prevention and management strategies to reduce complications [8,9]. 

This case report highlights an atypical presentation of PTS. The patient initially exhibited unilateral symptoms that progressively worsened over the course of a year, followed by acute involvement of the contralateral extremity. The symptom distribution was unique, with greater lower brachial plexus involvement and ascending paralysis. A lumbar puncture revealed albuminocytologic dissociation (ACD), which helped rule out other neuromuscular conditions and infections. The patient presented to the emergency department with a sudden loss of function in the right arm, without preceding trauma and with an unremarkable review of systems. An initial diagnosis of PTS in the left upper extremity (LUE) was made after nine months of extensive workup and specialist consultations. Fourteen months later, PTS was confirmed as the cause of the additional contralateral right upper extremity (RUE) symptoms. This unusual progression underscores the importance of timely diagnosis, targeted intervention, and a collaborative multidisciplinary approach to optimize outcomes and minimize long-term morbidity and complications.

## Case presentation

Patient information

A 53-year-old male with a medical history of hypertension, moderate bilateral cervical foraminal stenosis, and left-sided PTS presented to the emergency department with right index finger pain and weakness for two weeks. This new weakness followed a 14-month history of similar symptoms in his left arm, which began distally in the left index finger and progressively spread proximally until complete flaccid paralysis of the entire LUE. He denied any recent trauma, dizziness, blurred vision, dysarthria, dysphagia, or headache. The patient had been following up with neurosurgery and orthopedics departments.

Clinical findings

On physical examination, the patient was alert and oriented to person, place, and time. Cranial nerves were intact, with fluent speech and no evidence of aphasia or dysarthria. There was normal facial symmetry, hearing, symmetrical palate rise, and a midline tongue, with no atrophy or fasciculations. The gait was normal and steady, and there were no signs of truncal ataxia, with a negative Romberg test. Deep tendon reflexes were 1+ throughout, and both Hoffman’s sign and clonus were negative. Strength testing revealed significant new deficits in the RUE, which had developed acutely over the past two weeks, with 1/5 strength in finger abduction and flexion, 2/5 in wrist flexion and extension, and preserved strength (5/5) in elbow flexion and extension. This new symptom progression followed a 14-month history of LUE weakness. The LUE demonstrated complete flaccid paralysis, with 0/5 strength in the fingers, wrist, elbow, and shoulder, and significant atrophy of the proximal muscles more than the distal. This pattern of progressive weakness in the LUE had initially started distally in the left index finger and ascended to involve the entire upper extremity over time. Strength in both lower extremities (right lower extremity (RLE) and left lower extremity (LLE)) was normal (5/5) across all ranges of motion. Sensation to light touch was intact throughout, and proprioception was preserved in the bilateral lower extremities. 

**Figure 1:**
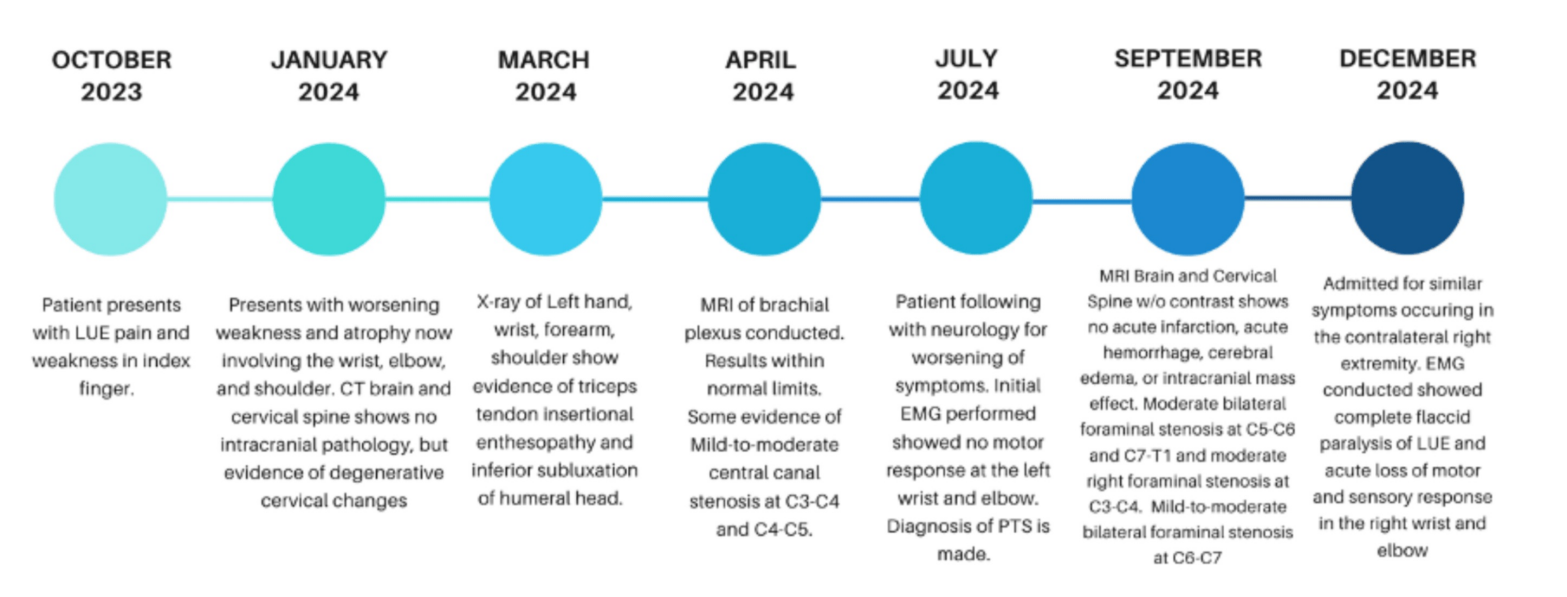
Clinical timeline and progression of PTS. This figure illustrates the chronological progression of symptoms and diagnostic findings in the patient with PTS over a 14-month period. Key milestones include the initial presentation of LUE pain and weakness, the development of worsening atrophy and flaccid paralysis, imaging studies (CT, MRI), and electrodiagnostic evaluations (EMG). The timeline highlights the eventual bilateral involvement and severe motor deficits leading to hospitalization and treatment. PTS: Parsonage-Turner syndrome; LUE: Left upper extremity; CT: Computed tomography; MRI: Magnetic resonance imaging; EMG: Electromyography; C4: 4th cervical vertebra; C5: 5th cervical vertebra; C6: 6th cervical vertebra; C7: 7th cervical vertebra; T1: 1st thoracic vertebra

The timeline and progression of the disease in the patient are presented in Figure 1.

Imaging and electrodiagnostic studies

**Figure 2:**
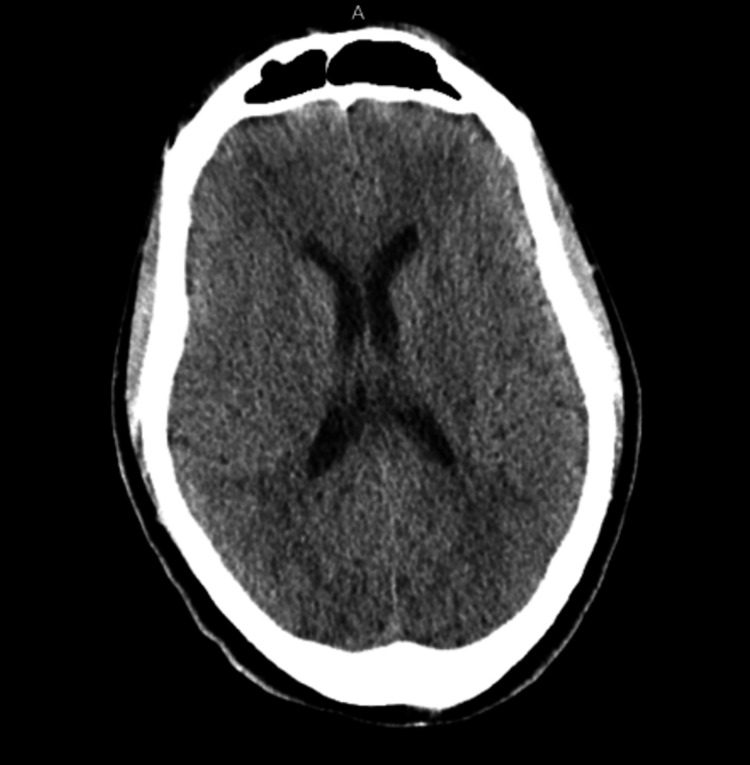
CT of brain without contrast showing no acute intracranial pathology. Ventricular and sulcal size and configuration were within normal limits. No acute loss of gray-white differentiation was noted, nor was there any acute intracranial hemorrhage or extra-axial collection. There was no evidence of mass effect or midline shift. The basilar cisterns were patent. There was no acute displaced calvarial fracture. The tympanomastoid air cells, along with the visualized portions of the orbits and paranasal sinuses, were within normal limits. CT: Computed tomography

**Figure 3:**
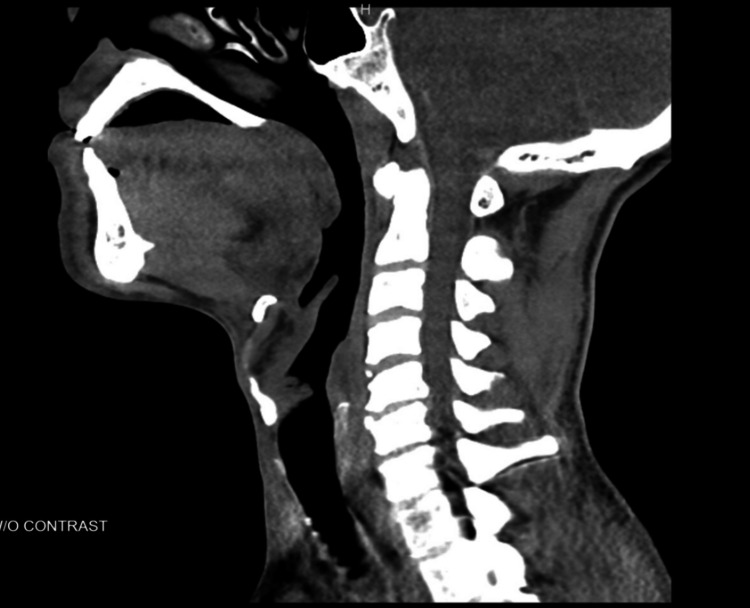
CT of cervical spine without contrast showing no acute fracture or subluxation. Cervical lordosis was maintained, and the posterior margins of the vertebral bodies were aligned. The vertebral bodies heights were maintained, with no evidence of atlantooccipital dislocation. No prevertebral soft tissue edema was noted. There were biapical blebs and multilevel degenerative changes of the cervical spine, characterized by varying degrees of disc space narrowing, marginal osteophytosis, endplate sclerotic or cystic changes, and facet hypertrophy. CT: Computed tomography

**Figure 4:**
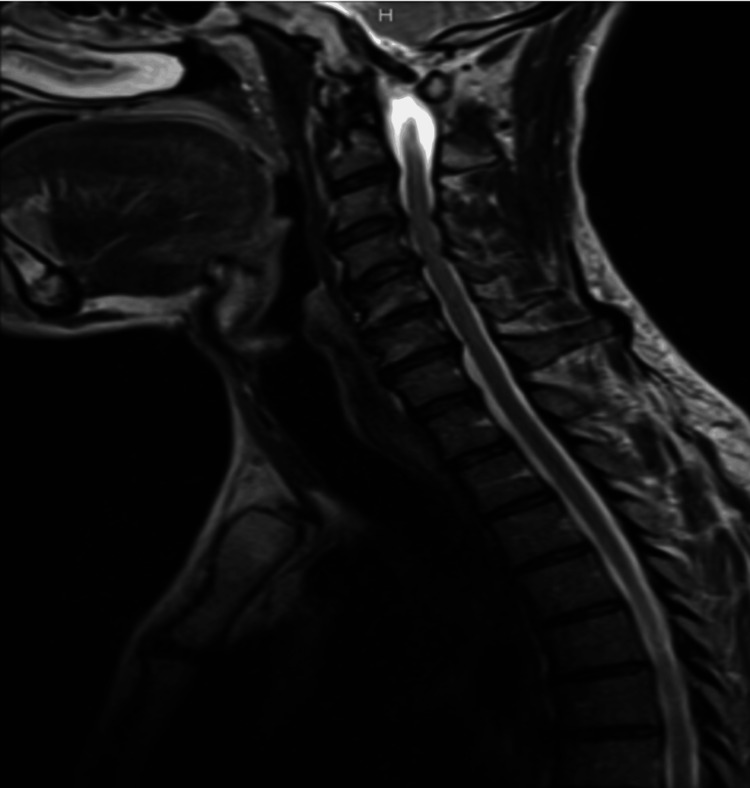
MRI of cervical spine indicating moderate bilateral foraminal stenosis at C5-C6 and C7-T1, with moderate right foraminal stenosis at C3-C4. Mild-to-moderate bilateral foraminal stenosis was observed at C6-C7. MRI: Magnetic resonance imaging; C3: 3rd cervical vertebra; C4: 4th cervical vertebra; C5: 5th cervical vertebra; C6: 6th cervical vertebra; C7: 7th cervical vertebra; T1: 1st thoracic vertebra

**Figure 5:**
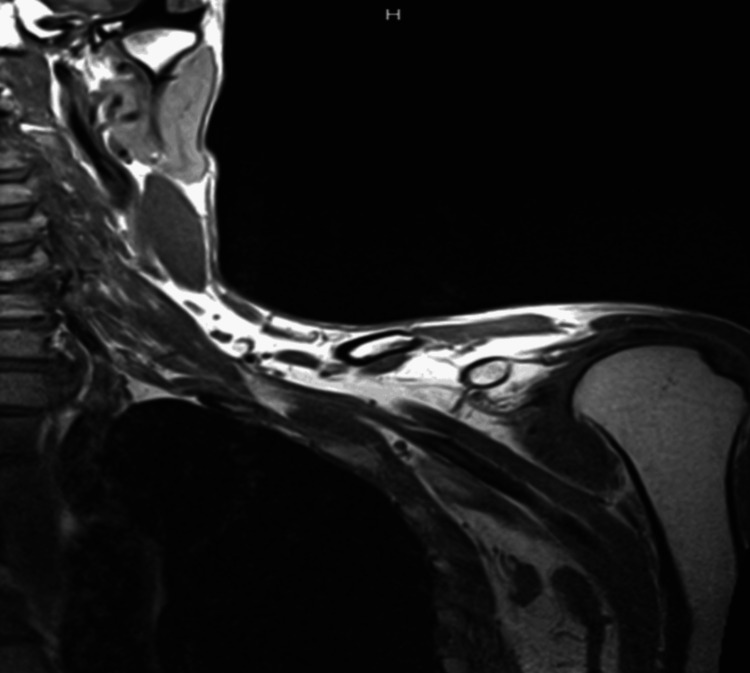
MRI of brachial plexus showing no T2 signal abnormality or regional abnormal enhancement. No invading mass or focal abnormality was observed in the lung apex or regional soft tissues. MRI: Magnetic resonance imaging

**Figure 6:**
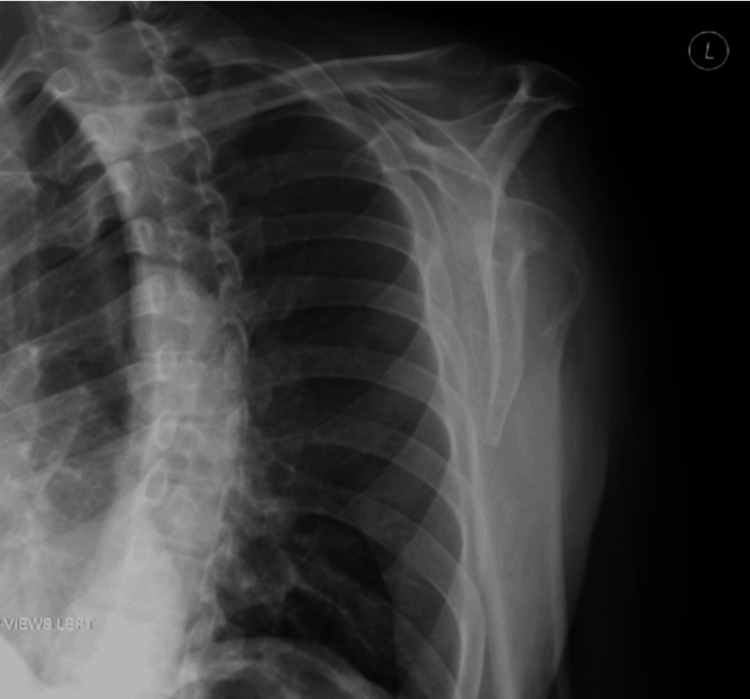
X-ray of LUE showing triceps tendon insertional enthesopathy and inferior subluxation of humeral head after significant proximal muscle atrophy. LUE: Left upper extremity

Imaging studies were performed to evaluate potential underlying causes. Computed tomography (CT) of the brain and cervical spine were negative for abnormalities (Figures 2-3). MRI of the cervical spine revealed moderate bilateral foraminal stenosis at 5th and 6th cervical vertebrae (C5-C6) and the 7th cervical vertebra and 1st thoracic vertebra (C7-T1), with moderate right foraminal stenosis at the 3rd and 4th cervical vertebrae (C3-C4) (Figure 4). MRI of the brachial plexus was unremarkable; however, central canal stenosis was confirmed at C3-C4 and C4-C5, consistent with findings from the cervical spine MRI (Figure 5). An additional X-ray of the left upper extremity showed triceps tendon insertional enthesopathy and inferior subluxation of the humeral head (Figure 6).

**Figure 7:**
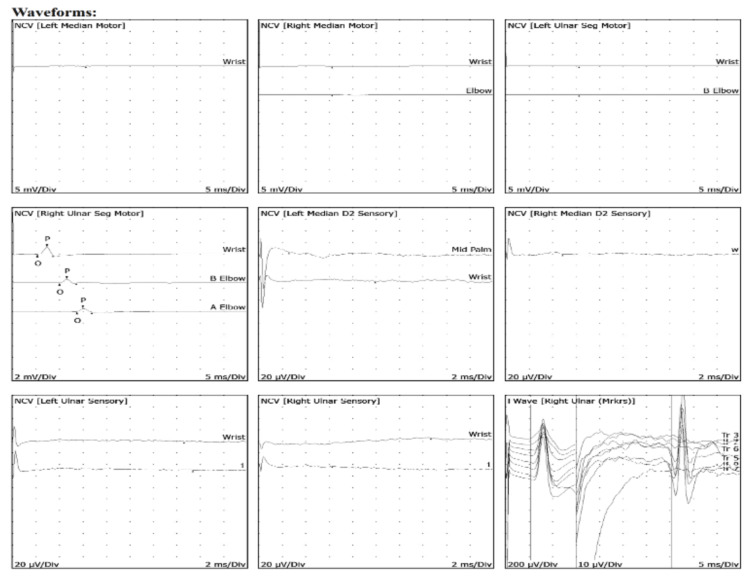
Waveforms from nerve conduction studies showing both motor and sensory axonal denervation. NCV: Nerve conduction velocity; D2: Index finger

Two trials of EMG and nerve conduction studies were conducted five months apart. The initial test for primary left-sided symptoms revealed no response of the left median motor nerve (wrist). The right median motor nerve showed prolonged distal onset latency (4.7 ms), reduced amplitude (3.2 mV), and decreased conduction velocity (elbow-wrist, 45 m/s). The second EMG study, conducted after the onset of right-sided symptoms, revealed no response in the left median motor nerve at the wrist. Similarly, the right median motor nerve demonstrated no response at both the wrist and elbow. The left ulnar motor nerve showed no response at the wrist and elbow, while the right ulnar motor nerve exhibited prolonged distal onset latency (5.3 ms), reduced amplitude (1.3 mV), and normal conduction velocity between the elbow and wrist (55 m/s) (Table 1). Sensory nerve studies indicated no response in the left median sensory nerve at the mid-palm and wrist, as well as in the right median sensory nerve at the wrist. Additionally, both the left and right ulnar sensory nerves showed no response at the wrist (Table 2). All F-wave latencies were within normal limits (Table 3). These findings suggested acute and chronic demyelinating neuropathy with significant functional impairments. The EMG revealed 2+ fibrillations and positive sharp waves in the first dorsal interosseous and extensor carpi radialis longus (Table 4). Additionally, the waveforms shown in Figure 7 confirmed the motor and sensory axonal denervation observed in the EMG and nerve conduction studies. 

**Table 1:** Motor nerve conduction studies summary NR: No response; O-P: Measurement of voltage difference between peak (P) and baseline (O) of muscle action potential; Delta-T: Width of highlighted selection; APB: Abductor pollicis brevis; ADM: Abductor digiti minimi

Nerve and Muscle	Stim Site	Response	Onset Latency (ms)	Normal Onset (ms)	O-P Amplitude (mV)	Normal O-P Amplitude (mV)	Negative Duration (ms)	Negative Area (mVms)	Segment	Delta-T (ms)	Distance (cm)	Velocity (m/s)	Normal Velocity (m/s)
Left Median Motor (APB)	Wrist	NR	—	<4.2	—	>4	—	—	—	—	—	—	—
	Elbow	NR	—	—	—	—	—	—	—	—	—	—	—
Right Median Motor (APB)	Wrist	NR	—	<4.2	—	>4	—	—	Elbow - Wrist	22	>50	—	—
	Elbow	NR	—	—	—	—	—	—	—	—	—	—	—
Left Ulnar Motor (ADM)	Wrist	NR	—	<3.4	—	—	—	—	Below Elbow - Wrist	0	>50	—	—
	Below Elbow	NR	—	—	—	—	—	—	—	—	—	—	—
Right Ulnar Motor (ADM)	Wrist	Present	5.3	<3.4	1.3	>4	3.28	1.81	Below Elbow - Wrist	4.7	26	55	>50
	Below Elbow	Present	10	—	0.7	—	3.59	0.97	Above Elbow - Below Elbow	3.8	14	37	—
	Above Elbow	Present	13.8	—	—	—	3.13	0.85	—	—	—	—	—

**Table 2:** Sensory nerve conduction studies summary NR: No response; O-P: Measurement of voltage difference between peak (P) and baseline (O) of muscle action potential; Delta-T: Width of highlighted selection; D2: Index finger; D4: Ring finger

Nerve and Site	Stim Site	Response	Onset Latency (ms)	Normal Onset (ms)	O-P Amplitude (mV)	Normal O-P Amplitude (mV)	Negative Area (mVms)	Segment	Delta-T (ms)	Distance (cm)	Velocity (m/s)	Normal Velocity (m/s)
Left Median Sensory (D2)	Mid Palm	NR	—	—	—	—	>20	Mid Palm - D2	7	—	—	—
	Wrist	NR	—	—	—	—	—	Wrist - Mid Palm	7	—	—	—
Right Median Sensory (D2)	Wrist	NR	—	—	—	—	>20	Wrist - D2	7	—	—	—
Left Ulnar Sensory (D4)	Wrist	NR	—	—	—	—	>18.0	Wrist - D4	14	—	>48.0	>48.0
Right Ulnar Sensory (D4)	Wrist	NR	—	—	—	—	>18.0	Wrist - D4	14	—	>48.0	>48.0

**Table 3:** Summary of F-wave studies L: Left; R: Right

Nerve and Muscle	F-Latency (ms)	Normal F-Latency (ms)	L-R F-Latency Difference (ms)	M-Latency (ms)	F-Latency - M-Latency (ms)
Right Ulnar (ADM)	35.27	<36	—	5.29	29.98

**Table 4:** Summary of EMG findings EMG: Electromyography; C6: 6th cervical spinal nerve; C7: 7th cervical spinal nerve; C8: 8th cervical spinal nerve; T1: 1st thoracic spinal nerve; nml: Normal; Fibs: Fibrillations; Psw: Positive sharp waves; Ins Act: Insertional activity; Amp: Amplitude; Dur: Duration; Poly: Polyphase; Recrt: Recruitment; Int Pat: Interval pattern; Dec: Decreased; Fasic: Fasciculations; Ext Car Rad: Extensor carpi radialis longus; 1st Dor Int: 1st dorsal interosseous

Side	Muscle	Nerve	Root	Ins Act	Fibs	Psw	Amp	Dur	Poly	Fasic	Recrt	Int Pat
Left	1st Dor Int	Ulnar	C8-T1	Dec	2+	2+	nml	nml	nml	0	nml	nml
Left	Ext Car Rad	Radial	C6-C7	Dec	2+	2+	nml	nml	nml	0	nml	nml

Laboratory studies

Laboratory studies performed upon admission, including a complete blood count (CBC), comprehensive metabolic panel (CMP), basic metabolic panel (BMP), lipid panel, fasting glucose, and hemoglobin A1c, were all within normal limits. Tests evaluating for potential infection and inflammation, including erythrocyte sedimentation rate (ESR), C-reactive protein (CRP), urinalysis (UA), and viral studies (varicella zoster virus, herpes simplex virus, West Nile virus, cytomegalovirus, lyme disease, *Toxoplasma gondii*, and syphilis serology (Venereal Disease Research Laboratory test (VDRL)), were also unremarkable (Table 5).

**Table 5:** Lumbar puncture results CSF: Cerebrospinal fluid; Ig: Immunoglobulin; PCR: Polymerase chain reaction; RBC: Red blood cell; WBC: White blood cell; VZV: Varicella zoster virus; HSV: Herpes simplex virus; VDRL: Venereal Disease Research Laboratory test (screen for syphilis, *Treponema pallidum*); AB: Antibodies

Test/Parameter	Patient Value	Reference Range/Units
Pre-centrifugation appearance, CSF	Bloody	—
Post-centrifugation appearance, CSF	Clear	—
RBC, CSF	16,050 /mcL	—
WBC, CSF	3 /mcL	0-5 /mcL
Glucose, CSF	62 mg/dL	50-80 mg/dL
Protein, CSF	79.0 mg/dL	15.0-60.0 mg/dL
Meningitis/encephalitis panel by PCR	Negative	Negative
West Nile virus AB IgM, CSF	<0.90	<0.90
West Nile virus AB IgG, CSF	<1.3	<1.3
*Toxoplasma gondii* IgG, CSF	<0.90	<0.90
VZV PCR, CSF	Not detected	Not detected
HSV 1 DNA, CSF	Not detected	Not detected
HSV 2 DNA, CSF	Not detected	Not detected
Lyme AB IgG, CSF	Not detected	Not detected
Lyme AB IgM, CSF	Not detected	Not detected
VDRL, CSF	Non-reactive	Non-reactive
Oligoclonal bands, CSF	Absent	Absent (≤1 band normal)

A lumbar puncture revealed ACD, characterized by an elevated cerebrospinal fluid (CSF) protein level of 79 mg/dL with a normal white blood cell count (3 cells/mm³). The CSF red blood cell count was significantly elevated (16,050 cells/mm³), most likely due to a traumatic tap. After centrifugation, the CSF sample result turned clear. Despite thorough testing, no evidence of active infection or inflammatory processes was identified. Further endocrine and nutritional evaluations, including vitamin B12, thyroid-stimulating hormone (TSH), iron studies, and lactate levels, were all within normal limits. Hematologic and coagulation panels, including prothrombin time/international normalized ratio (PT/INR) and activated partial thromboplastin time (aPTT), were also unremarkable, ruling out coagulopathies or hematologic abnormalities as contributors to the clinical presentation.

Therapeutic intervention

Over a 14-month period marked by progressive ascending weakness, atrophy, and flaccid paralysis of the LUE, the patient did not receive pharmacological treatment due to the chronic nature of the condition. Upon hospital admission following the acute involvement of the contralateral RUE, the patient was empirically treated with IVIG at a dose of 52.8 g once daily for five days. The rationale for IVIG treatment was based on the acute contralateral involvement and the presence of ACD, suggesting a potential autoimmune and inflammatory etiology. To mitigate potential infusion-related adverse effects such as nausea, fluid overload, renal, and cardiac complications, the patient was closely monitored and premedicated with oral acetaminophen (650 mg) and diphenhydramine (25 mg) prior to each IVIG infusion. Physical and occupational therapy (PT/OT) was initiated at a frequency of 45 minutes per day for five consecutive days during the hospital stay. Following completion of the IVIG treatment and rehabilitation, the patient's motor strength improved to 3/5 in the RUE. The patient was subsequently discharged with recommendations for outpatient follow-up, including repeat EMG, neurology evaluation, and laboratory testing for anti-ganglioside antibodies (GM1, GD1a, GD1b). Additionally, weekly PT/OT sessions were recommended to address ongoing functional impairments.

## Discussion

PTS presents a diagnostic challenge due to its non-specific presentation and overlap with other musculoskeletal and neurological disorders. This case describes a 53-year-old male patient with a unique presentation of PTS, initially suspected to have cervical radiculopathy secondary to foraminal stenosis based on imaging findings. However, the absence of a dermatomal pain pattern, progressive LUE weakness, and EMG findings ultimately led to a diagnosis of PTS.

Initial EMG, conducted nine months after symptom onset, revealed severe axonal damage and demyelination. Notably, no motor response was detected in the left median nerve, while the right median motor nerve showed a delayed onset latency of 4.7 m/s and decreased amplitude of 3.2 mV. Abnormal spontaneous activity, including moderate fibrillations and positive sharp waves, was observed in muscles innervated by the left median, ulnar, and radial nerves. Fibrillation potentials are spontaneous discharges from individual muscle fibers due to denervation, suggesting ongoing or recent nerve injury. Positive sharp waves, often occurring alongside fibrillations, indicate muscle membrane instability due to denervation or myopathy. While mild axonal damage was noted in the right median nerve, the patient remained asymptomatic on the right side at this time, with preserved 5/5 motor strength. In contrast to cervical radiculopathy, where fibrillations and positive sharp waves are typically accompanied by increased motor unit action potential amplitudes, polyphasia, and reduced recruitment due to reinnervation, the EMG findings in this case pointed more toward PTS. Additionally, in cervical radiculopathy, sensory nerve action potentials (SNAPs) often remain intact, as the lesion is typically proximal to the dorsal root ganglion, and pain usually precedes a dermatomal pattern of weakness. In PTS, however, weakness and pain coexist, as seen in this patient’s presentation. Given these unique EMG findings and the absence of structural abnormalities on imaging, cervical radiculopathy was considered less likely and PTS was deemed a more probable diagnosis [10,11].

Progressive weakness in the LUE, leading to flaccid paralysis and atrophy, prompted a repeat EMG three months later. The second study demonstrated worsening axonal loss in the LUE, with no recordable motor potentials in the median, ulnar, and radial nerves. Motor unit recruitment was severely reduced in proximal and distal muscles, consistent with ongoing denervation. The study also revealed new abnormalities in the RUE, including absent motor responses in the median and ulnar nerves and reduced recruitment patterns in the intrinsic hand muscles. These findings indicated contralateral progression, highlighting the patchy yet asymmetric nature of motor deficits in PTS.

The presence of ACD during lumbar puncture was an unexpected finding in this case, raising initial concerns for Guillain-Barré syndrome (GBS) or chronic inflammatory demyelinating polyneuropathy (CIDP). ACD, characterized by elevated CSF protein without leukocytosis, is traditionally associated with these immune-mediated polyneuropathies. However, in this patient, the clinical presentation of acute, asymmetric, patchy upper extremity motor deficits, rather than symmetric ascending weakness in the lower extremities typical of GBS, strongly supported PTS as the underlying diagnosis. ACD in PTS may result from localized brachial plexus inflammation or disruption of the blood-nerve barrier, leading to protein leakage into the CSF. Further research is warranted to explore the pathophysiological role of ACD in PTS and its potential as a distinguishing feature from other neuropathies [12].

Some cases of PTS showed improvement with off-label IVIG and adjunct glucocorticoid use in acute cases; however, there is little evidence for its efficacy in chronic situations. Another article suggested that in acute PTS with severe pain, patients can be given opioids, tricyclic antidepressants, and anti-epileptic drugs [13]. In our case, empirical IVIG was administered due to the presence of ACD and concern for inflammatory neuropathy. IVIG led to partial improvement in motor strength in RUE, increasing strength in the third and fourth digits from 2/5 to 3/5 by the time of hospital discharge. However, the treatment did not improve the chronic flaccid paralysis in LUE, likely due to the prolonged duration of symptoms prior to intervention. The findings underscore the importance of early recognition and intervention in minimizing irreversible motor deficits.

The pathophysiology of PTS remains incompletely understood, though it is believed to be an autoimmune or inflammatory condition, often triggered by viral infections, trauma, vaccinations, pregnancy, chemotherapy, and radiation. In this case, the absence of recent infections, trauma, or vaccinations, and the prevention of worsening symptoms with IVIG suggests an idiopathic or autoimmune etiology. The patient's lack of significant improvement over 14 months prior to IVIG treatment suggests a more chronic form of PTS, emphasizing the variable course of this condition [14].

Management of PTS remains primarily supportive, focusing on pain control, rehabilitation, and functional support. In this case, the patient benefited from a multidisciplinary approach, including neurology follow-up, physical and occupational therapy, and bracing for shoulder subluxation and wrist instability. The patient's left shoulder and wrist instability were likely due to the atrophy caused by chronic PTS. Stabilization of RUE symptoms with IVIG and occupational therapy suggests that early multidisciplinary care may halt disease progression.

This case underscores the importance of maintaining a broad differential diagnosis for acute upper extremity weakness and considering PTS, particularly in the absence of clear structural or traumatic causes (Table 6) [15]. EMG studies are pivotal in establishing the diagnosis, as they provide evidence of axonal denervation, demyelination, and patchy asymmetric motor deficits. A collaborative approach involving neurology, immunotherapy, and rehabilitation is essential for optimizing recovery. This case also highlights the importance of recognizing atypical presentations of PTS (Table 7), including bilateral expansion and the presence of ACD, which can inform diagnostic and management strategies for this rare and often misunderstood condition. 

**Table 6:** Differential diagnoses of PTS PTS: Parsonage-Turner syndrome; GBS: Guillain-Barré syndrome; CRPS: Complex regional pain syndrome; M: Male; F: Female; ELISA: Enzyme-linked immunosorbent assay; CSF: Cerebrospinal fluid; ROM: Range of motion; US: Ultrasound; CT: Computed tomography; MRI: Magnetic resonance imaging; EMG: Electromyography Credits: Created by the authors

Category	Condition	Key Symptoms	Diagnostic Features	At-Risk Population	Risk Factors
Neuromuscular disorders	PTS	Sudden-onset, patchy neuropathic pain followed by delayed brachial plexus weakness; asymmetric atrophy; irregular nerve involvement	MRI: muscle edema, hourglass constrictions; EMG: denervation in a non-dermatomal pattern; CSF: albuminocytologic dissociation	Young to middle-aged adults, M>F	Viral illness, vaccination, autoimmune and inflammatory disease, idiopathic
	Multifocal motor neuropathy	Painless, asymmetric distal weakness without sensory loss	EMG: conduction block, anti-GM1 antibodies	Middle-aged males	Autoimmune predisposition
	Mononeuritis multiplex (vasculitic)	Asymmetric, painful neuropathies affecting multiple nerves	EMG: axonal damage; nerve biopsy: vasculitis	Adults with systemic vasculitis	Autoimmune disease, hepatitis B/C
	GBS	Rapidly progressive weakness, classically ascending; can involve upper limbs; areflexia	CSF: albuminocytologic dissociation; EMG: demyelination	All ages, post-infectious	Campylobacter infection, recent illness
Cervical and spinal pathology	Cervical radiculopathy (degenerative)	Gradual neck pain with radiating arm pain, paresthesia, weakness in dermatomal distribution	Positive Spurling’s test; MRI: foraminal stenosis	Middle-aged adults	Spondylosis, disc degeneration
	Cervical radiculopathy (disc rupture)	Acute neck/arm pain, dermatomal motor/sensory loss	MRI: disc herniation; positive Spurling’s test	Younger adults (30-50 years)	Lifting injury, acute neck trauma
	Cervical spondylosis with brachialgia	Activity-dependent neck pain, no clear dermatomal pattern	X-ray: spondylotic changes; MRI: cervical degeneration	>50 years, sedentary adults	Aging, repetitive strain
Orthopedic and shoulder conditions	Rotator cuff pathology	Shoulder pain, weakness in abduction/external rotation	Positive impingement tests (e.g., Neer’s, Hawkins, drop arm); MRI: tendon pathology	>40 years, overhead athletes	Overuse, aging, trauma
	Adhesive capsulitis	Severe pain progressing to stiffness, loss of external rotation	Clinical: active and passive ROM restriction; MRI: capsular thickening	Middle-aged adults, F>M	Diabetes, hypothyroidism, immobility
	Subacromial bursitis	Nighttime pain, tenderness over lateral shoulder, painful arc (60-120°)	US: bursal fluid	Overhead workers, athletes	Overuse, inflammatory arthritis
	Calcific tendinitis	Intermittent flare-ups of severe shoulder pain, stiffness	X-ray: calcium deposits; US: hyperechoic deposits	30-50 years old	Overuse, metabolic issues (e.g., diabetes)
	Thoracic outlet syndrome	Pain, paresthesia, worsens with overhead activity, possible vascular signs (pulse loss)	Dynamic tests (Roos, Adson’s); MRI/US for vascular/nerve compression	Young adults, overhead athletes	Extra cervical rib, scalene hypertrophy
Autoimmune and systemic causes	CRPS	Diffuse pain, swelling, vasomotor instability, progressive weakness	Bone scan: asymmetric uptake; clinical diagnosis	Post-injury/surgery	Fracture, nerve trauma
	Focal motor neuron disease	Slowly progressive, painless weakness without sensory involvement	EMG: motor neuron dysfunction	Adults >50 years	Unknown
Infectious and post-infectious causes	Lyme disease	Intermittent limb weakness, migratory pain, erythema migrans rash, fatigue	ELISA and Western blot for Borrelia	Endemic regions, outdoor workers	Tick exposure
	Asian tick-borne encephalitis	Viral prodrome - severe headache, back pain, acute flaccid shoulder paralysis	CSF: pleocytosis; MRI: anterior horn involvement	Endemic regions (Asia)	Tick exposure, travel history
Oncologic and rare causes	Scapular tumors (primary)	Localized scapular pain/mass, possible scapular winging	X-ray, MRI, CT: abnormal bone lesion; biopsy confirms	Any age	Prior malignancy, bone tumor history
	Entrapment neuropathies	Pain, weakness, sensory deficits if mixed nerve compressed	EMG: conduction block; MRI/US: nerve compression	Overhead athletes, post-surgical patients	Overuse, trauma

**Table 7:** Key findings and case report summary LUE: Left upper extremity; RUE: Right upper extremity; MRI: Magnetic resonance imaging; EMG: Electromyography; WBC: White blood cell; CSF: Cerebrospinal fluid; IVIG: Intravenous immunoglobulin; PTS: Parsonage-Turner syndrome; GBS: Guillain-Barré syndrome; C3: 3rd cervical vertebra; C4: 4th cervical vertebra; C5: 5th cervical vertebra; C6: 6th cervical vertebra; C7: 7th cervical vertebra; T1: 1st thoracic vertebra

Category	Findings	Remarks
Clinical presentation	LUE 0/5 strength in the fingers, wrist, elbow, and shoulder. RUE 1/5 strength in finger abduction and flexion, 2/5 in wrist flexion and extension, and preserved strength (5/5) in elbow flexion and extension.	Initial LUE involvement and complete flaccid paralysis; subsequent contralateral progression in right extremity.
	No dermatomal pain distribution, nor exacerbated by neck positioning; pain and weakness disproportionate to physical findings; no inciting event.	Suggestive that symptoms are unlikely due to cervical radiculopathy or stenosis.
Imaging studies	MRI of brain and cervical spine unremarkable for acute pathology.	Findings ruled out structural causes (e.g., radiculopathy).
	Cervical foraminal stenosis noted (C3-C4, C5-C6, C7-T1).	Not consistent with clinical progression or symptoms.
	Brachial plexus MRI: No abnormalities detected.	No focal mass or compression ruled out as a cause of symptoms.
EMG	Initial: Severe axonal damage; patchy denervation; absence of motor response in LUE.	Confirmed acute and chronic demyelinating neuropathy.
	Follow-up: Progression to RUE axonal denervation.	Highlighted patchy, asymmetric nature of deficits.
CSF analysis	Albuminocytologic dissociation (protein: 79 mg/dL; WBC: 3 cells/mm³).	Elevated protein suggests inflammatory or autoimmune involvement.
	No infectious causes detected.	—
Treatment	IVIG therapy initiated following bilateral involvement.	Partial recovery in RUE strength; no improvement in chronic LUE paralysis.
	Multidisciplinary approach: Physical therapy, occupational therapy, bracing.	Stabilized progression; optimized rehabilitation.
Pathophysiology	Likely autoimmune etiology; no recent infections or vaccinations.	Emphasized idiopathic nature in this case.
Unique features	Bilateral progression over 14 months.	Rare in typical PTS presentation.
	Albuminocytologic dissociation with upper extremity ascending paralysis.	Distinguishes from structural or infectious neuropathies (GBS).

## Conclusions

This case highlights the diagnostic complexity and clinical variability of PTS, emphasizing the importance of maintaining a broad differential diagnosis when evaluating acute upper extremity weakness. The idiopathic and atypical presentation in this patient, including bilateral progression, severe axonal and demyelinating neuropathy, the presence of ACD in CSF, and improvement of acute symptoms with IVIG therapy underscores the necessity of a thorough diagnostic approach incorporating clinical evaluation, imaging, and electrodiagnostic studies. EMG played a pivotal role in identifying patchy, asymmetric motor deficits and confirming the diagnosis of PTS, distinguishing it from other conditions such as cervical radiculopathy and inflammatory polyneuropathies like GBS or CIDP. The presence of ACD and treatment with IVIG, though unusual in PTS, provides a potential avenue for future research into the inflammatory and autoimmune mechanisms underlying this condition. Management of PTS remains largely supportive, with an emphasis on pain control, physical and occupational therapy, and targeted interventions. Early recognition and treatment may help mitigate disease progression and improve outcomes, though challenges persist in managing chronic forms of PTS.
